# Marginal fit and fracture resistance of CAD/CAM glass ceramic occlusal veneers with different preparation designs

**DOI:** 10.1186/s12903-025-05889-4

**Published:** 2025-05-25

**Authors:** Suad M. Hassan, Ayat G. Montaser, Zinab R. El Sharkawy, Nevin A. Gad, Shaimaa A. Alrafee, Zahraa A. Gabal

**Affiliations:** 1 Crowns and Bridges, Faculty of Dental Medicine for Girls, Al-Azhar University, Cairo, Egypt; 2 Operative Dentistry, Faculty of Dental Medicine for Girls, Al-Azhar University, Cairo, Egypt

**Keywords:** Different preparation designs, IP E.max CAD, Occlusal veneer, Marginal fit, Fracture resistance

## Abstract

**Background:**

To assess marginal fit and fracture resistance of CAD/CAM glass ceramic occlusal veneers with different preparation designs.

**Methods:**

First premolar typodont maxillary was chosen. Standard IPS E.max CAD occlusal veneer preparations were carried out using Exocad software using three distinct designs: the first design involved a minimally invasive preparation (butt joint"BJ"group); the second design involved an occlusal veneer preparation with a circumferential hollow chamfer finish line"HC"group; and the third design involved a deep chamfer finish line"DC"group. To create a total of 24 epoxy resin replicas, each prepared design was reproduced eight times ("*n* = 8"for each prepared design). Every sample was made using IPS E.max CAD ceramics. Every occlusal veneer was firmly attached to the matching epoxy resin using adhesive resin cement. A computerized stereomicroscope was used to measure the vertical marginal gap. Ultimately, the fracture resistance was measured using a universal testing apparatus.

**Results:**

Deep chamfer occlusal veneer design, group"DC", registered statistically significant the highest mean value of vertical marginal gap (118.38 ± 10.43 μm) as well as the lowest mean value of failure load (549.97 ± 56.66 N). While butt joint occlusal veneer design"BJ"registered a statistically significant lowest mean value of vertical marginal gap (99.2 ± 7.15 μm) as well as the highest mean value of failure load (1107.25 ± 93.09 N).

**Conclusions:**

Although different preparation designs of IPS E.max CAD occlusal veneer restorations would significantly affect the marginal fit and fracture resistance, all groups were within the clinically accepted range.

## Background

Dental professionals frequently use the concepts of tooth preparation in conjunction with a fully covered crown for restoring lost tooth structure and improving occlusal force resistance. But with intact teeth structural tissues, these parameters may be overly intrusive as they need reduction of whole coronal surface, which affects occlusal vertical dimension, jawbone connection, and occlusal stability [1].

Consequently, the preservation of the healthy tooth structure is essential for the lifespan of teeth and restorations. Since the main principles of biomimetic dentistry is minimizing the removal of the healthy tooth structure, which aims to strengthen the tooth while minimizing the risk of future decay or damage [2].

Therefore, partial coverage restorations-like those with ceramic occlusal veneers-have become a prevalent treatment option for posterior teeth with coronal damage either due to caries or non-caries issues as abfraction [3]. It has been shown that new adhesive occlusal veneer designs, such as the hollow chamfer (HCD) and proposed modified design (PMD), have been effective for less invasive indirect occlusal restorations depending on clinical circumstances [4]. HCD involves hollow chamfer prep at the occlusal third, making it suited for both lasting and cosmetic restorations, while PMD involves deep chamfer prep at the occlusal third [4]. These designs are a consequence of the development of adhesive materials, which have led to a shift in focus from mechanical retention to adhesive and biomimetic techniques [5].

Lithium disilicate is a fascinating contemporary material related to this advancement. Lithium disilicate's biomechanical qualities make it suitable for application in the posterior region with a minimum thickness of 0.7 mm, without compromising strength. This makes it the preferred material for those novel occlusal veneer preparation designs [6, 7].

The degree of marginal fit is a major determinant of how long indirect restorations last. Recurrent cavities, luting cement deterioration, microleakage, and restorative failure can all be caused by marginal inaccuracy [8]. Fractures in all ceramic restorations are the most frequent reason for these restorations'failure. Furthermore, the characteristics of the underlying supporting structures, such as their elastic modulus, the thickness of all ceramic restorations, or the preparation design, may have an impact on the fracture resistance of occlusal veneers [9].

It’s worth mentioning that only a limited number of studies have investigated the impact of innovative hollow chamfer preparation design on the characteristics of occlusal veneer restorations, with most of the other conventional designs focusing mainly on the fracture resistance of the restorations [1, 7]. Most of the previous pertinent research has assessed inlays and/or onlays that provide partial cusp coverage [10].

An information gap exists concerning the impact of novel hollow chamfer preparation configuration on the marginal fit and fracture resistance of ceramic occlusal veneers. Therefore, this research was directed towards evaluating the aforementioned design with those of conventional designs of occlusal veneer. According to the null hypothesis, different preparation designs would not significantly affect the marginal fit and fracture resistance of CAD/CAM glass ceramic occlusal veneer restorations.

## Materials and method

### Study design

The study followed an in vitro, parallel-controlled design in which three parallel groups’ marginal fit and fracture resistance were assessed. In order to have sufficient power to execute a hypothesis test, a power analysis was created. The estimated sample size (N) was a total of 24 samples (i.e., *n* = 8 samples per group) by adopting an alpha (α) level of 0.05, a beta (β) level of 0.2) (i.e. power = 80%), and an effect size (d) of 1.24 computed based on the results of prior studies [1, 3].The G Power software version (3.1.9.4) was used to calculate the sample size.

To focus on the research question in the current study and facilitate data interpretation, PICO, which involves 4 elements: problem (P), intervention (I), control (C), and outcome (O), was addressed as follows:

- P: Defected porous, weak enamel with caries extension (problem).
- I: Hollow chamfer and proposed modified design of occlusal veneers (intervention).
- C: Butt joint design of occlusal veneer (control).
- O: Protection of the weak cusps and elimination of carious lesions with an esthetic way out (outcome).

### Construction of 3D printed dies

**Fig. 1:**
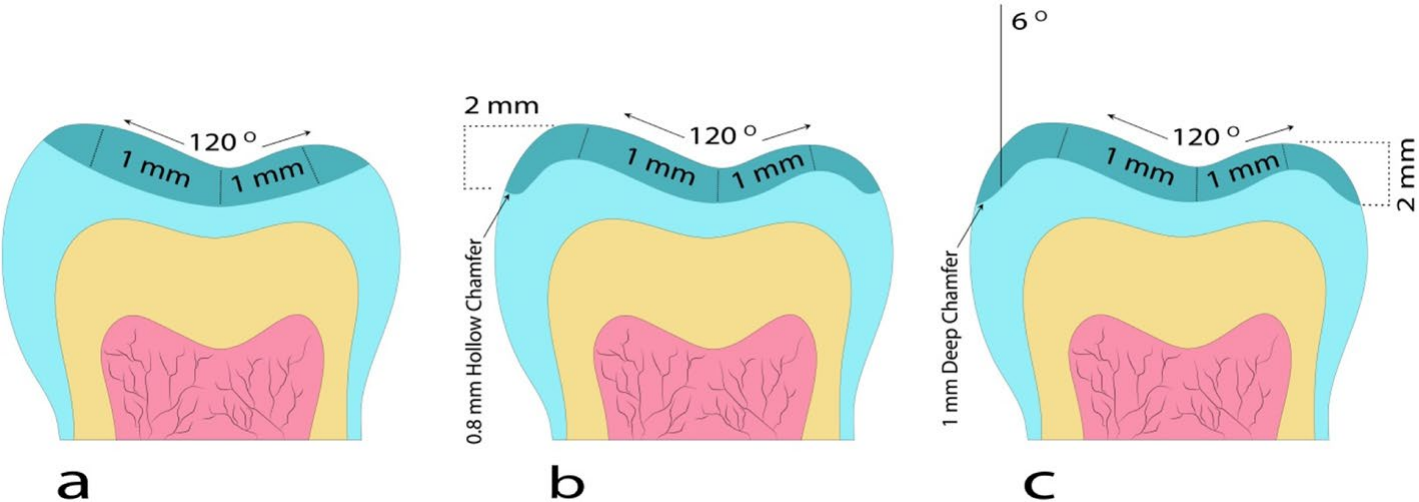
Occlusal veneer preparation designs; **a** Butt joint design. **b** Hollow chamfer design. **c** Proposed modified design

Three typodont maxillary first premolars (Nissin Dental Product, Inc., Japan) with the same dimensions were collected. 3D printing technology was used to perform the standard preparations of occlusal veneers, according to the following steps: The teeth were sprayed with a light-reflecting powder and then scanned (Medit i500, MEDIT CORP, Korea) to make an optical impression. Data were transferred to Exocad computer program software (Exocad Software GmbH, Germany) to design standard preparations of occlusal veneers as follows:

- Group (BJ): Minimally invasive preparation of occlusal veneer with anatomical occlusal reduction 1 mm (butt joint design) [11] (Fig. 1A).
- Group (HC): Occlusal veneer with anatomical occlusal reduction 1 mm extended on the axial walls, 2 mm apical to the prepared occlusal surface terminating with a 0.8 mm circumferential hollow chamfer finish line (hollow chamfer design) [12] (Fig. 1B).
- Group (DC): Occlusal veneer with anatomical occlusal reduction of 1 mm and a 1 mm circumferential deep chamfer finish line (proposed modified design) [11] (Fig. 1C).

After that, the designs were saved as a standard tessellation language (STL) file. This file was transferred to a 3D printer machine (ANYCUBIC Photon Mono SE, China) to print the resin teeth, which possess a modulus of elasticity comparable to dentin (12.9 GPa).

### Manufacturing and duplication of epoxy resin dies

**Fig. 2:**
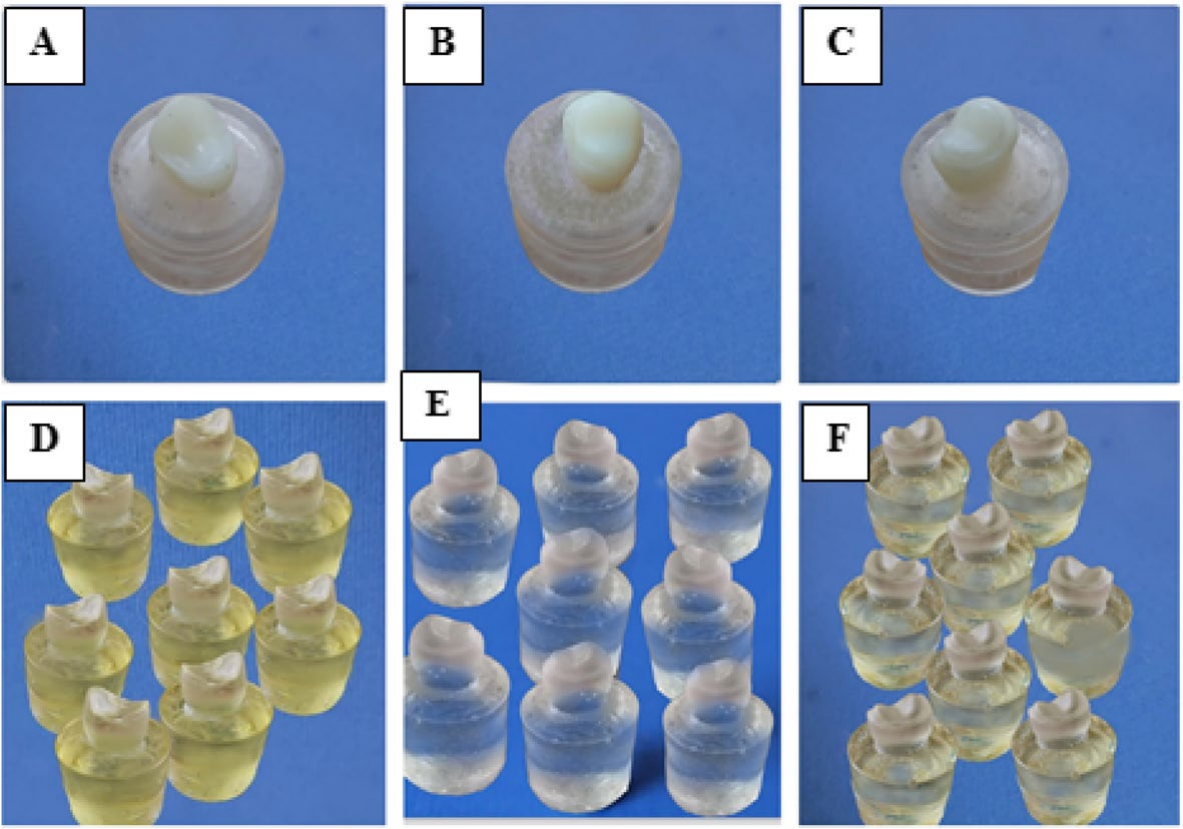
Manufacturing and duplication of epoxy resin dies; **A** Prepared 3D-printed butt joint design embedded in epoxy resin. **B** Prepared 3D-printed hollow chamfer design embedded in epoxy resin. **C** Prepared 3D-printed proposed modified design embedded in epoxy resin. **D** Epoxy resin dies for butt joint design. **E** Epoxy resin dies for hollow chamfer design. **F** Epoxy resin dies for proposed modified design

Each prepared 3D-printed resin die was inserted into the middle of a plastic cylinder, filled with epoxy resin (EgyPro epoxy resin, EgyPoxy, Egypt), leaving 2 mm below the cervical line, simulating the bone level. After complete polymerization of epoxy resin (24 h), the blocks were removed from the plastic cylinders, smoothed, and finished (Fig. 2 A-C). The silicon mold for the prepared 3D-printed teeth was made using duplicating addition silicon material (Replisil 22, Dent-e-con, Germany). Each preparation was duplicated 8 times by means of epoxy resin to give a total of twenty-four epoxy resin dies (Fig. 2 D-F).

### Sample grouping

A total of twenty-four epoxy dies were used in the present study. Epoxy dies were divided into three groups according to different preparation designs used for the fabrication of occlusal veneers; group (BJ): 8 epoxy dies with minimally invasive preparation (butt joint design) (control group), group (HC): 8 epoxy dies with hollow chamfer design and group (DC): 8 epoxy dies with deep chamfer finish line (proposed modified design).

### Occlusal veneer fabrication

All occlusal veneers were fabricated from IPS E.max CAD as follow: With the Medit i500 scanner (Medit i500, MEDIT CORP, Korea), digital imprint was created. Then occlusal veneers were designed using Exocad software (Exocad software GmbH, Germany) with a spacer setting of 50μm and the margins for digital dies have been determined and drawn. After determining the line of insertion, milled material was chosen, which included IPS E.max CAD blocks (Ivoclar Vivadent, Liechtenstein, Germany) to begin planning the restoration. Then milling was performed using a Roland system (Roland DG Corporation, Japan). After that, all occlusal veneers were glazed using FLUO Ivocolor glaze paste (Ivoclar Vivadent, Liechtenstein). The crystallization process was conducted in strict accordance with the manufacturer’s instructions, utilizing a Programat P310 furnace (Ivoclar Vivadent AG, Schaan/Liechtenstein), then all occlusal veneers were checked on their corresponding dies.

### Bonding of occlusal veneers

The following steps were used to surface treat each occlusal veneer's intaglio surface [9]: A) Hydrofluoric acid (9.5% concentration) is applied for 10 s. B) Water and dryness were used to rinse the fitting surface until a chalky look was verified. C) Applying a silane coupling agent to the fitting surface for 60 s, then letting it dry, silanizes it.

"SuperCem"resin cement was used to cement each occlusal veneer on its corresponding die in compliance with the manufacturer's instructions. Each occlusal veneer was kept under a 3 kg static load until the cement had completely set using a specially made cementation apparatus [13]. The cementation process began with the vertical bar sliding lower until it made contact with the seated restoration. The materials and equipment used in this study are listed in Tables 1 and 2.

**Table 1:** Used materials in this study

Material	Product	Composition	Manufacture
Lithium disilicate glass–ceramic	IPS E.max CAD	SiO_2_-Li_2_O,K_2_O,ZnO,P_2_O_5_, Al _2_O_3_, ZrO_2_	Ivoclar Vivadent
Bonding agent	SuperCem adhesive resin cement	- Resin Matrix as Bis-GMA and UDMA - Fillers: inorganic particles as silica or glass - Self-Adhesive Monomers: as HEMA and MDP	DentKist
Hydrofuoric acid gel	Porcelain Etch	% 9.5 HF	Bisco, Inc
Porcelain Primer	Silane coupling agent	- Inorganic-reactive group: methoxy, ethoxy - Organic-reactive group: silane	Bisco, Inc

Sio_2_ Silicon dioxide, Li_2_O Lithium oxide,K_2_O Potassium oxide, ZnO Zinc oxide, Al _2_O_3_ Aluminium oxide, ZrO_2_ Zirconium oxide, Bis-GMA bisphenol A glycidyl methacrylate, UDMA Urethane dimethacrylate, HEMA hydroxyethyl methacrylate, MDP 10-methacryloyloxydecyl dihydrogen phosphate, HF Hydrofuoric acid

**Table 2:** Used equipments in this study

Equipment	Product	Manufacture
Extra-oral scanner	Medit i500	Medit Corp
Furnace	Programat furnace (P300*)*	Ivoclar Vivadent

### Testing procedures

#### Marginal fit determination

Using a digital stereomicroscope (MA100 Nikon stereomicroscope, Japan), the vertical marginal gap distance was measured in order to assess the marginal fit. Twelve points were measured on the dies, three on each surface, with a set spacing between the points. Photographs of margins were taken at a fixed 50X magnification. The gap width was measured and assessed using a computerized image analysis system (OmniMet image analysis software).

#### Fracture resistance

Through the recording of sample failure under compressive force, fracture resistance was ascertained. The stainless-steel ball with a diameter of 4 mm was fixed to the upper moveable head of the universal testing machine (Instron model 3345, Norwood, USA) and used to apply a constant static force (5 KN) to dies until the specimen fails. An axial compression force is delivered at a crosshead speed of 1.0 mm/min to the center of the occlusal surface. After that, failure mode analysis was executed using a magnifying lens (10 ×) to classify the failure mode according to Burk's classification [14]:

- Code I: Minimal fracture or crack in occlusal veneer.
- Code II: Less than half of occlusal veneer is lost.
- Code III: Occlusal veneer fracture through the midline (half of the occlusal veneer is displaced or lost).
- Code IV: More than half of occlusal veneer is lost.
- Code V: Sever fracture of the epoxy resin die and/or the occlusal veneer.

### Statistical analysis

Statistical analysis was performed using the Statistical Package for Social Sciences (SPSS) version 20 for Windows, Chicago, IL, USA. Normality was checked using Kolmogorov–Smirnov and Shapiro–Wilk tests. Marginal fit and fracture resistance were summarized using mean and standard deviation, confidence intervals and range. ANOVA test was used to analyze data between groups, followed by Bonferroni post hoc for pairwise comparisons. All a-values are two-sided. A-values ≤ 0.05 were considered significant. Pearson correlation test was used to measure the strength of the linear relationship between two variables. It has a value between − 1 to 1, with a value of − 1 meaning a total negative linear correlation, 0 being no correlation, and + 1 meaning a total positive correlation.

## Results

### Results of marginal fit (vertical marginal gap distance “μm”)

**Fig. 3:**
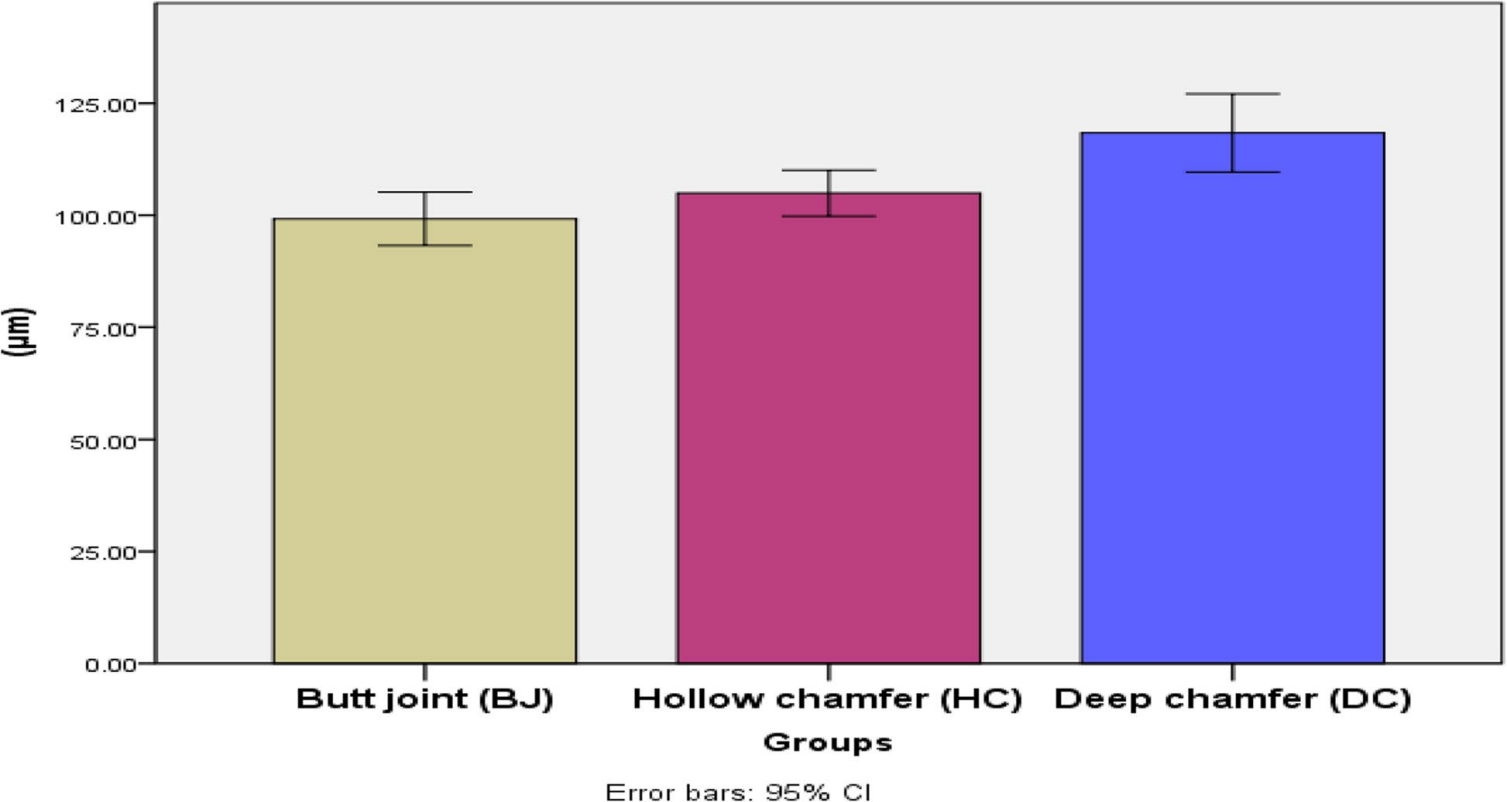
Bar chart illustrating mean marginal fit in different preparation designs used for the fabrication of occlusal veneers

**Fig. 4:**
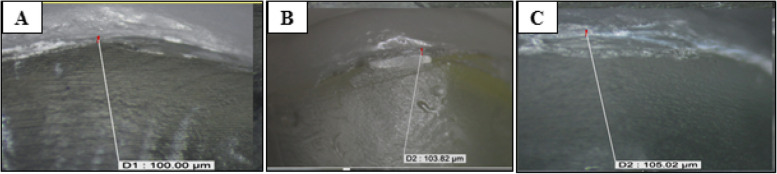
Digital microscopic images of vertical marginal gap distance at different occlusal veneer designs; **A** Butt joint design. **B** Hollow chamfer design. **C** Proposed modified design

The highest value was recorded in deep chamfer, group (DC) (118.38 ± 10.43μm), followed by hollow chamfer, group (HC) (104.92 ± 6.15μm), with a significantly lowest value recorded in butt joint, group (BJ) (99.2 ± 7.15μm). The difference between groups was statistically significant (*P* < 0.001). Post hoc test used for pairwise comparison revealed no significant difference between hollow chamfer (HC) and deep chamfer (DC) (Table 3, Fig. 3). Stereomicroscopic images of vertical marginal gap distances at different groups are presented in (Fig. 4):

**Table 3:** Descriptive statistics and comparison of marginal fit (µm) in different preparation designs used for the fabrication of occlusal veneers (ANOVA test)

	**Preparation** **designs**	**Mean**	**Std. Dev**	**95% Confidence Interval for Mean**		**Min**	**Max**	**F**	***P* value**
				Lower Bound	Upper Bound				
**Marginal** **fit**	**Butt joint (BJ)**	99.20 ^b^	7.15	93.23	105.18	89.20	109.83	11.77	.000*
	**Hollow chamfer (HC)**	104.92^b^	6.15	99.77	110.07	97.71	114.90		
	**Deep chamfer (DC)**	118.38^a^	10.43	109.66	127.10	100.61	130.20		

Significance level *p* ≤ 0.05, * significant

Post hoc test: within the same comparison, means sharing the same superscript letter are not significantly different

### Results of fracture resistance (failure load “N”)

**Fig. 5:**
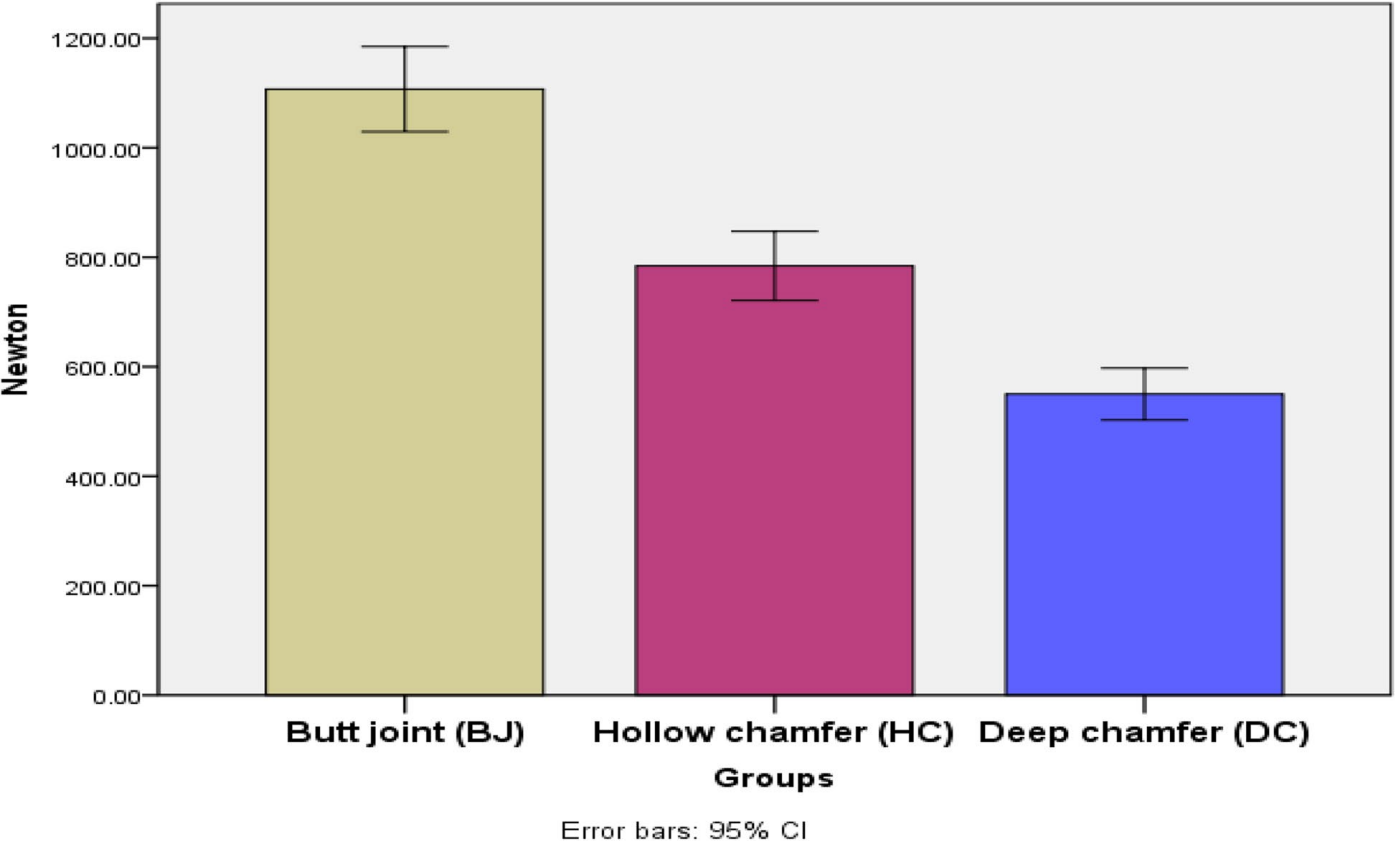
Bar chart illustrating mean fracture resistance in different preparation designs used for the fabrication of occlusal veneers

The highest value was recorded in butt joint, group (BJ) (1107.25 ± 93.09 N), followed by hollow chamfer, group (HC) (784.23 ± 75.47 N), with a significantly lowest value recorded in deep chamfer,group (DC) (549.97 ± 56.66 N). The difference between groups was statistically significant (*P* < 0.001). Post hoc test used for pairwise comparison revealed a significant difference between each 2 groups (Table 4, Fig. 5).

**Table 4:** Descriptive statistics and comparison of fracture resistance (Newton) in different preparation designs used for the fabrication of occlusal veneers (ANOVA test)

	**Preparation** **designs**	**Mean**	**Std. Dev**	**95% Confidence Interval for Mean**		**Min**	**Max**	**F**	***P* value**
				Lower Bound	Upper Bound				
**Fracture** **resistance**	**Butt joint (BJ)**	1107.5^a^	93.09	1029.43	1185.08	986.71	1232.84	106.94	.000*
	**Hollow chamfer (HC)**	784.23^b^	75.47	721.14	847.32	707.96	897.23		
	**Deep chamfer (DC)**	549.97^c^	56.66	502.60	597.35	493.31	634.97		

Significance level *p* ≤ 0.05, * significant

Post hoc test: within the same comparison, means sharing the same superscript letter are not significantly different

**Fig. 6:**
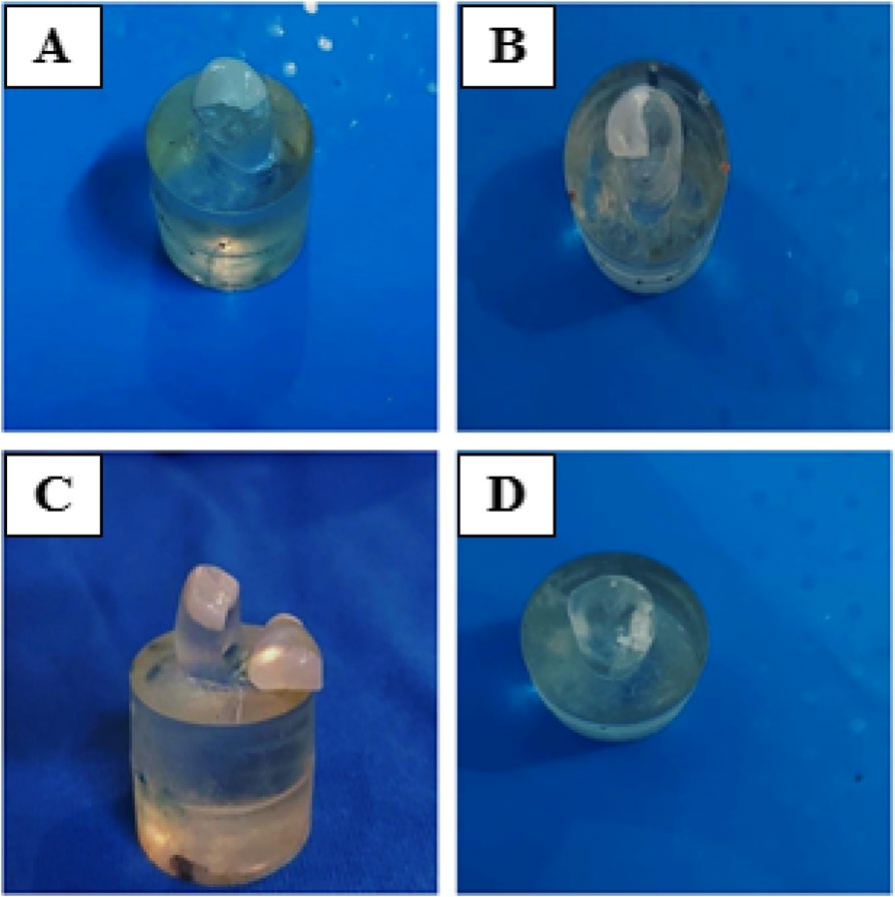
Failure mode; **A** Occlusal veneer fracture through midline (half of the veneer is displaced or lost) (Code III). **B** Fracture of more than half of the occlusal veneers (Code IV). **C**, **D** Sever fracture of epoxy resin die or occlusal veneer (Code V)

**Fig. 7:**
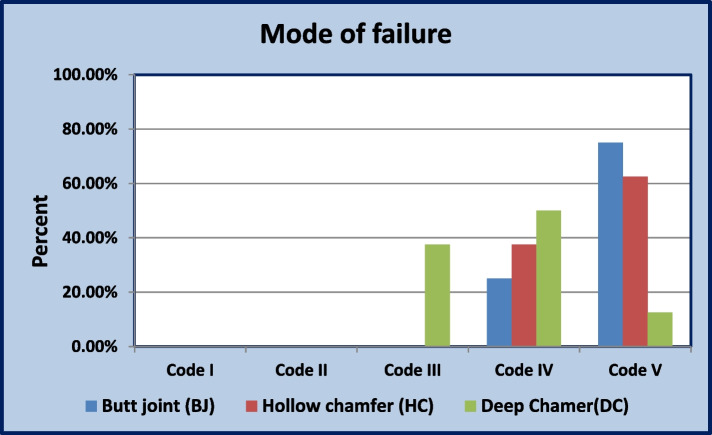
Bar chart illustrating mode of failure between groups

Regarding to mode of failure: Chi square test revealed that the difference between groups was statically significant (*P* < 0.001). In the group (BJ and HC) (*n* = 8), the most common failure mode was (code V) (*n* = 6"75%") (*n* = 5"62.5%") respectively, followed by (code VI) (*n* = 2"25%") (*n* = 3"37.5%") respectively, with no sample recorded failure mode with (Codes I, II, and III). While in the group (DC) (*n* = 8), the most common failure mode was (code IV) (*n* = 4"50%"), followed by (code III) (*n* = 3"37.5%) and (code V) (*n* = 1"12.5%") (Figs. 6 and 7).

### Correlation between marginal fit and fracture resistance

**Fig. 8:**
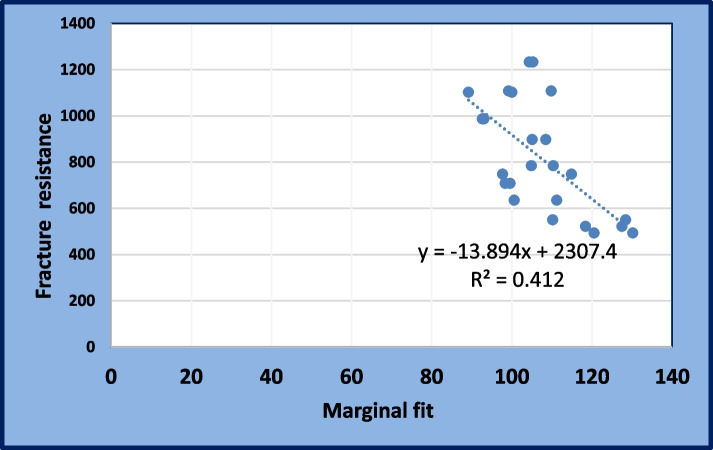
Scatter plot showing correlation between fracture resistance and marginal fit

In butt joint (BJ), a strong positive correlation was noted between marginal fit and fracture resistance. However, this correlation was not statistically significant (*p* = 0.087). But, in hollow chamfer (HC), a weak positive correlation was noted between marginal fit and fracture resistance. However, this correlation was not statistically significant (*p* = 0.357). On the other side, in deep chamfer (DC), a strong negative correlation was noted between marginal fit and fracture resistance. This correlation was statistically significant (*p* = 0.027). Overall, a strong negative correlation was noted between marginal fit and fracture resistance. This correlation was statistically significant (*P* < 0.001) (Table 5 and Fig. 8).

**Table 5:** Correlation between marginal fit and fracture resistance (Pearson’s correlation test)

**Groups**		**Correlation**
**Butt joint (BJ)**	Pearson Correlation (R)	.641
	*P* value	.087 ns
	Interpretation	Strong positive
**Hollow chamfer (HC)**	Pearson Correlation	.378
	*P* values	.357 ns
	Interpretation	Weak positive
**Deep chamfer (DC)**	Pearson Correlation	-.766-^*^
	*P* value	.027
	Interpretation	Strong negative
**Overall**	Pearson Correlation	-.642-^**^
	*P* value	.001
	Interpretation	Strong negative

## Discussion

The main difficulty in restorative dentistry is finding least invasive preparatory methods that yield outstanding cosmetic results while maintaining tooth integrity. Reducing tooth preparation encourages the maintenance of the dento-enamel junction and tooth structure, which are important for stress redistribution, preventing cracks from spreading, and enhancing tooth durability [15].

Numerous dental ceramics with varying chemical structures and technological processing have been proposed and recommended to satisfy patients'cosmetic expectations as well as functional and mechanical requirements. With single restorations in particular, the advancements in material research with CAD/CAM technology have allowed dentists to prepare teeth more conservatively, removing less tooth structure in the process [16].

Restorations fabricated of glass ceramic as IPS e.max CAD still have the best translucency and cosmetics to this day. In addition, it offers fast fabrication times and standard thickness. Additionally, the mechanical qualities of this restoration type are greatly improved by the adhesive approach used [17].

A difference in preparation design is essential for assessing the marginal fit and fracture resistance of indirect restorations, consequently affecting the clinical outcome and long-term prognosis of ceramic restorations [3, 4]. In order to give the grantee the best standardization and high accuracy of the preparation design with regard to the amount of reduction, anatomical features, and finish line, as well as to avoid many technical laboratory and clinician variables, 3D printing technology was utilized in the current study to create physical models for the three designs [9]. In the current study, three preparation designs were chosen for occlusal veneer preparation. The first was the butt joint design, which is a more conservative preparation that is dictated by the anatomy and a 1 mm occlusal reduction. Current studies suggest that the thickness of all-ceramic occlusal veneers can be reduced to 0.5–1 mm while the restoration can still withstand typical masticatory stresses [18, 19]. The second with a hollow chamfer finish line, which promotes a smooth transition between the finish line area and internal line angle, providing more excellent cosmetics [20]. While the third has a deep chamfer finish line, increasing the amount of enamel as the margin shape cuts enamel prisms nearly parallel to their longitudinal axis, it creates more favorable circumstances for the adhesion process [20].

To create epoxy resin blocks, each prepared 3D printed design was inserted in a plastic container that was filled with epoxy resin material. Natural teeth would exhibit significant variation based on age, anatomy, and storage period following extraction, which can make standardization challenging. For this reason, epoxy resin dies were utilized in the current investigation in place of natural teeth [7]. Conversely, the epoxy resins die exhibits great transverse strength, abrasion resistance, and superior dimensional accuracy with surface detail replication. Furthermore, it possesses modulus elasticity comparable to dentin (12.9 GPa) [21].

Comparing data on marginal adaptation from various studies poses challenges and potential inaccuracies due to several factors, including variations in preparation design, measurement techniques, the number and location of measurement points, the type of resin cement employed, and the method used to fabricate the restoration [22, 23]. Advanced 4- or 5-axis milling machines, which can enhance the fit between the prepared tooth structure and the restoration’s intaglio surface, also contribute to these variations. However, the precision and reliability of the outcomes improve when more measurement points are included and consistently assessed at the same locations across samples. This approach reduces variability, ensuring that the results accurately reflect the restoration’s adaptation quality. Our study adopted this approach to ensure accurate and reliable results. Therefore, carefully considering these variations is essential when making in vitro comparisons of data [22, 23].

In this research, the restorations were adhered under 3 kg applied weight to standardize the pressure applied [13]. However, to ensure consistency in the cementation procedure, all restorations were placed by the same operator. Dual-cured adhesive resin cement was used to bond occlusal veneers in order to provide controlled working time and ensure that the polymerization process is completed in the event that light is insufficient [24]. Adhesively cemented monolithic ceramic restorations have been shown to have a substantially greater fracture resistance than those made with traditional cementation [25].

One of the key factors determining a dental restoration's long-term viability is marginal fit. Leakage can result from an inadequate marginal fit, which can lead to micro leakage, luting agent dissolution, and carious lesions, among other problems. It may also affect how durable the repair is and its capacity to tolerate functional loading. Since a digital stereomicroscope is thought to be a non-invasive research tool, it was utilized in this study to examine and assess the marginal gap [3].

The outcomes of the current research refute the null hypothesis by indicating that changes to the occlusal veneer design can have a major impact on the marginal gap and failure load.

Based on the findings in the current study for registered vertical marginal gap distance mean values of IPS e.max CAD ceramic occlusal veneers, Table 3 and Fig. 2; deep chamfer, group (DC) registered the highest significant value of marginal gap (118.38 ± 10.43 μm) followed by hollow chamfer, group (HC) (104.92 ± 6.15 μm), with a significantly lowest value recorded in butt joint, group (BJ) (99.2 ± 7.15 μm). As reported in ELsharkawy et al. [8], all groups fell within the clinically authorized variety of 40–120µm.

These findings are similar to those of Falahchai et al. [1] and Hasan and Abdul-Ameer [11] who estimated the marginal fit of overlays with different preparation designs and reported that butt-joint design recorded the lowest marginal gap distance. These findings could be explained by the fact that the amplitude of the marginal gap increased when the designs changed to more complicated or retentive geometries [26]. Moreover, it has been proposed that the simple preparation features of the butt joint design, such as flat, smooth occlusal reduction, lack of retentive features, and fewer internal angles, have been suggested to facilitate all digital workflow processes by enabling milling burs to replicate restoration details, ultimately lowering the marginal gap [3].

Conversely, these findings contradict those of Emam and Aleem [9], Shalaby and Abo-Eittah [26], who all concluded that the modified prepared designs of occlusal veneer with a finish line recorded better marginal fit than conventional butt joint designs with no finish line. These findings could be attributed to many factors, including variations in preparation designs, repair manufacturing materials, and gap measurement techniques. Furthermore, a smaller marginal gap was achieved because the modified occlusal veneer design with a finish line provides more support than the traditional design with an indefinite finish line, which offers more surface area for bonding and good internal adaptation for the restoration [27].

Additionally, the margin of butt joint preparation might have thin areas, which raises the possibility of chipping during manufacture. The CAD/CAM milling machines require milling burs with a minimum margin thickness of 0.3 mm for precise milling. This can be challenging to achieve in the butt joint preparation design, leading to a larger vertical marginal gap distance [28].

Another crucial element that was thought to determine how long all-ceramic restorations would last clinically was their failure load. Dental glass ceramics are naturally brittle; therefore, they have excellent resistance to compressive loads. However, they are very susceptible to tensile and shear stresses, which might lead to restorative failure during clinical service [29].

According to reports, occlusal strength varies according to age, gender, and the tooth's strategic location in the arch. In terms of clinical practice, the usual biting force for the maxillary premolar area is between 222 and 445 N, which reaches about 800 N during clenching [30, 31]. The three preparation designs in the current investigation had mean fracture loads that were higher than the range of realistic occlusal forces in the premolar area. So, all samples should be able to tolerate the highest intraoral masticatory forces [30, 31].

Based on the findings in the current study for registered fracture resistance in the current study, Table 4 and Fig. 4; butt joint (group BJ) registered the highest significantly mean value of failure load. These findings were in concurrence with Huang et al. [31] and Elassy et al. [32], all concluding that veneers with only occlusal coverage recorded higher fracture resistance than veneers with occlusal and axial surfaces coverage. This could be explained based on the fact that more invasive axial wall preparation may result in higher tensile stresses inside the ceramic repair. Consequently, restoring more axial walls raises the maximum core stress in the restoration and decreases fracture resistance [31].

However, the results of the current study are in contrast to Emam and Aleem [9] and Halim [33], who revealed that the modified occlusal veneer design showed a higher fracture resistance mean value than the conventional design. This might be due to the modified design's advantage of having a circumferential finish line that evenly distributes stresses over the tooth. An enamel collar with external bevels surrounding a preparation improves occlusal force redirection along the tooth's longitudinal axis, potentially improving fracture behavior [34].

It is important to note that failure mode analysis adds clinical value to in-vitro studies and provides a valuable tool to estimate the restorability of a tooth following restoration failure. Catastrophic failure which included severe fracturing of occlusal veneers and epoxy resin dies was the most frequent failure pattern in more than half of the restoration in the current study (Figs. 5 and 6). This observation is consistent with the results documented in previous studies by Johnson et al. [35] and Alberto et al. [36], suggesting that these restorations are as strong as the worn teeth they are supposed to restore. Additionally, the efficient bonding between die and glass ceramics enables the force to transmit through the restoration-die complex [37].

The results of the correlation between marginal fit and fracture resistance revealed that there was a strong negative relation between the two variables (Fig. 7). This was similar to Angerame et al. [38], who revealed that the fracture resistance of partial indirect restoration does not appear to be primarily influenced by the marginal fit. They explained this finding by the location of the point of loading during the fracture test, which was not near the finishing line [34].

There were certain limitations to the current study. For example, the artificial aging process did not include any thermo-mechanical stress, which would have shed light on the detrimental impacts of the loading on the attributes under examination. Another drawback of the current study is that various different types of CAD/CAM ceramics should be compared. Furthermore, internal adaptability and horizontal marginal gap were not taken into account while evaluating the vertical margin gap. In addition, even when loads are delivered in multiple directions in clinical settings, only one compressive load per fracture was taken into account. Future studies should consider including an ageing procedure to evaluate the longevity of the obtained results. In addition, in vivo studies are recommended to validate the reliability of using occlusal veneers with different preparation designs and glass ceramic materials.

## Conclusions

According to the results of this research:

- 1. Preparation designs are important factors which influence their marginal fit and fracture resistance.
- 2. Marginal fit and fracture resistance of CAD/CAM lithium disilicate occlusal veneers are within the clinically accepted ranges.
- 3. Butt joint and hollow chamfer preparation designs of CAD/CAM lithium disilicate occlusal veneers have superior marginal fit and fracture resistance compared to proposed modified design.

## Data Availability

All data generated or analyzed during this study are included in this published article.

## Abbreviations

CAD/CAM: Computer aided design/computer aided manufacture
BJ: Butt joint
HCD: Hollow chamfer design
DC: Deep chamfer
PMD: Proposed modified design
3D: Three-dimensional
